# Exploring the role of managers in the development of a safety culture in seven French healthcare facilities: a qualitative study

**DOI:** 10.1186/s12913-020-05331-1

**Published:** 2020-06-08

**Authors:** Jean-Luc Quenon, Anthony Vacher, Marc Faget, Marie Levif-Lecourt, Tamara Roberts, Isabelle Fucks, Myriam Promé-Visinoni, Christine Cadot, Jean-Yves Bousigue, Bruno Quintard, Pierre Parneix, Catherine Pourin

**Affiliations:** 1 Comité de Coordination de l’Évaluation Clinique et de la Qualité en Nouvelle Aquitaine - Hôpital Xavier Arnozan, Allée du Haut-Lévêque, 33604 Pessac, France; 2 Institut de recherche biomédicale des armées - Unité d’Ergonomie cognitive des situations opérationnelles, 1 place Valérie André, 91223 Brétigny sur Orge, France; 3 Department of Operations Management, KEDGE Business School, 680 Cours de la Libération, 33405 Talence, France; 4 Département Management des Risques Industriels, 1 avenue du Général de Gaulle, 92141 Clamart, France; 5 Institut pour une culture de sécurité industrielle, 6 Allée Emile Monso, 31400 Toulouse, France; 6 Centre Hospitalier d’Agen, 21 Route de Villeneuve, 47923 Agen, France; 7 Laboratoire EA 4136 ‘Handicap, Activité, Cognition, Santé’, Université de Bordeaux, Faculté de Psychologie, 3 ter, place de la Victoire, 33076 Bordeaux, France; 8 Centre d’appui pour la Prévention des Infections Associées aux Soins de Nouvelle-Aquitaine, CHU de Bordeaux, Hôpital Pellegrin - Bâtiment Le Tondu, 33076 Bordeaux, France

**Keywords:** Healthcare facility, Manager, Qualitative study, Patient safety, Professional role, Safety culture

## Abstract

**Background:**

Numerous studies have been conducted over the past 15 years to assess safety culture within healthcare facilities; in general, these studies have shown the pivotal role that managers play in its development. However, little is known about what healthcare managers actually do to support this development, and how caregivers and managers represent managers’role. Thus the objectives of this study were to explore: i) caregivers and managers’ perceptions and representations of safety, ii) the role of managers in the development of safety culture as perceived by themselves and by caregivers, iii) managers’ activities related to the development of safety culture.

**Methods:**

An exploratory, multicentre, qualitative study was conducted from May 2014 to March 2015 in seven healthcare facilities in France. Semi-structured interviews were conducted with managers (frontline, middle and top level) and caregivers (doctors, nurses and nurse assistants) and on-site observations of two managers were carried out in all facilities. A thematic analysis of semi-structured interviews was performed. Observed activities were categorised using Luthans’ typology of managerial activities.

**Results:**

Participants in semi-structured interviews (44 managers and 21 caregivers) expressed positive perceptions of the level of safety in their facility. Support from frontline management was particularly appreciated, while support from top managers was identified as an area for improvement. Six main categories of safety-related activities were both observed among managers and regularly expressed by participants. However, caregivers’ expectations of their managers and managerial perceptions of these expectations only partially overlapped.

**Conclusions:**

The present study highlights current categories of managerial activities that foster safety culture, and points out an important gap between caregivers’ expectations of their managers, and managerial perceptions of these expectations. The findings underline the need to allow more time for managers and caregivers to talk about safety issues. The results could be used to develop training programs to help healthcare managers to understand their role in the development of safety culture.

## Background

Safety culture (SC) has been the focus of numerous studies over the past two decades, with the aim of using it as a lever to improve patient safety in healthcare facilities [1]. Many have focused on evaluation, with the development of often time-consuming and difficult-to-implement qualitative methods based on interviews and observations [2, 3], or faster and less-costly quantitative approaches [4]. The latter are typically based on questionnaires that measure the safety climate, i.e. the shared perceptions of members of an organisation regarding their work environment and the organisation’s safety policies [5–7].

There is no universally-accepted model of SC [8]. Two, classical, but contrasting approaches can be distinguished [9–11]. The first is a functionalist model [11]. This adopts a top-down, normative and unifying representation of organisational SC. As it is the expression of organisational ideology, goals and strategy, it tends to be seen as amenable to management control. The second is an interpretive, or anthropological model [9]. Here, SC is seen as a complex phenomenon that emerges from interactions between all actors, their internal and external relationships, the organisation and its environment. With this bottom-up approach, managers are only one actor among many others in the development of SC. Recently, Guillaume [12] proposed an integrated model of SC which acknowledges the importance of both a top-down and a bottom-up model of SC, as they complement each other.

The constituent dimensions of SC vary across studies [5, 7] and are the subject of debate [13]. Nevertheless, there seems to be a certain degree of consensus on the dominant influence of the organisation [14], leadership [15] and management [16–18]. Strenghtening the role of managers is one of the levers that has been identified as a way to improve the SC, which is still underdeveloped in healthcare facilities [19, 20].

In France, several studies have recurrently found that “management support for patient safety” is a poorly developed dimension of SC despite its importance [7, 21, 22].

Managers’ perceptions and representations of their activities and role in SC are crucial in fostering their commitment and actions [23]. However, few studies have focused on their role [24–27]. The notion of the ‘manager’ itself remains poorly understood [28] while, at the same time, little is known about how healthcare managers influence SC [29].

A manager is often defined as a professional who is responsible for all or part of the facility; his responsibilities include, coordination, organisation, planning and monitoring in order to achieve the facility’s goals and objectives [30]. He or she can operate at one of three levels: frontline management, middle management and top management [31]. Following Mintzberg [30] and direct observations of managers at work, the job has been described in terms of a set of activities or ‘roles’ – defined as organised sets of behaviours associated with a position – rather than as a well-defined profession.

In the healthcare domain, little is known about managers’ actual activities, in particular those intended to improve patient safety [29]. Therefore, the objectives of this study were to explore: i) caregivers and managers’ perceptions and representations of safety, ii) the role of managers in the development of safety culture as perceived by themselves and by caregivers, iii) managers’ activities related to the development of safety culture.

## Methods

### Definition and conceptual models of safety culture

Numerous definitions of SC have been proposed in the academic literature [8]. In the present study, we adopt the European Society for Quality in Health Care’s definition, which was specifically developed for the healthcare setting [32], namely, “an integrated pattern of individual and organisational behaviour, based upon shared beliefs and values that continuously seeks to minimise patient harm, which may result from the processes of care delivery” (p. 4).

In an integrated view of SC [12], the two classical, and contrasting models of safety culture (interpretative and functionalist models) were selected for our qualitative study.

### Study design, settings

An exploratory, multicentre, qualitative study was conducted from May 2014 to March 2015 in seven healthcare facilities in southwestern France. These facilities had agreed to participate following a request from the Aquitaine Regional Centre for Quality and Safety in Health Care*.* Three (facilities A, E, and F) were public hospitals with over 300 beds, three were private clinics with over 100 beds (facilities B, C and D), and one (facility G) was a private clinic with fewer than 100 beds. Five provide acute (medical, surgical and obstetric) care and two facilities (F and G) provide psychiatric care. One voluntary care unit participated per facility.

### Data collection

In each facility, two qualitative methods were applied. The first was a one-hour, semi-structured individual interview with managers at each level (four top managers, two middle managers where there were any, and two frontline managers) and three caregivers (a doctor, a nurse and a nursing assistant). All interviews were recorded and transcribed in full. Transcripts were not returned to participants for comment or correction. The interview guide addressed perceptions of safety within the facility, representations of roles, managerial activities with respect to safety, and caregivers’ expectations of their managers in terms of safety. This interview guide specifically designed for this study is provided in Additional file 1 and examples of questions extract from it are presented in Table 1. This approach made it possible to identify managerial expectation and activities attributed to managers by themselves and by caregivers they managed. Interview guides and questions were tested in a pilot study in a healthcare facility that was not part of the sample.

**Table 1:** Examples of questions from the semi-structured interview guide

Examples of questions contained in the semi-structured interview guide	
• Professional background: Did your training include elements related to safety?	
• Perception of safety: How would you rate the level of safety in your institution (excellent/good/acceptable/poor/unacceptable)?	
• Safety actions: What is your role in the development and application of safety rules, the facility safety policy, and more broadly, in safety management?	
• Support from hierarchy: Do you value compliance with safety rules and caregivers’ initiatives in terms of safety?	
• Safety expectations: In your opinion, what are caregivers’ expectations of their managers in terms of safety?	
• Socio-demographic data: age, seniority in the institution	

The second method involved direct, one-day observations of two managers at different levels in each facility. The day was chosen with the manager; it had to included, a period of time dedicated to safety issues. The manager was instructed not to change his agenda and to work as he usually does. Activities related to safety were assessed on the following criteria: context, interactions with other professionals, safety messages, and attitudes of caregivers. A case report form was used to record managers’ activities, which were structured into the following categories adapted from categories of effective managerial activities given in Luthans [17]: planning/ coordinating, staffing, training/ developing, decision-making/ problem solving, processing paperwork, exchanging routine information, monitoring/ checking performance, motivating/ reinforcing, disciplining/ punishing, interacting with outsiders, managing conflict and socialising/ politicking.

In each facility, both semi-structured interviews and on-site observations were carried out by one of two experienced sociologists (MLL and TR, with six- and five-years’ experience respectively) who also had experience in quality and safety research projects.

### Data analysis

Interview transcripts were subject to a thematic analysis using NVivo 10 qualitative data analysis software (QSR International Pty Ltd. Version 10, 2012). Data were encoded by the two sociologists (MLL and TR), based on the themes identified in the review of the academic literature and the content of interviews. The two sociologists worked together on coding in order to develop the analysis, and regularly consulted each other.

Observations were subject to a categorical analysis by the two sociologists using the same software as for the semi-structured interviews. The categorisation was based on the classification of managerial activities given in Luthans [17].

### Ethical considerations

Participation in the study was voluntary and informed consent was obtained from all participants before enrolment. To ensure anonymity, all identifying information was removed from participants’ responses.

## Results

### Participants

In the seven healthcare facilities, 65 professionals (37 women, 28 men) participated in semi-structured interviews. Table 2 shows their sociodemographic characteristics. Depending on the facility, the number of participants ranged from 8 to 11, including 3 caregivers and 5 to 8 managers. Most participants (83%) had worked in their facility for 5 years or more. Over two-thirds (70%) were 40 years of age or older, and more than half (57%) had risk management training. Among these 65 professionals, 44 (68%) were managers and 21 (32%) were caregivers (8 doctors, 6 nurses and 7 nursing assistants). Of the 44 managers, 27 were top managers (8 directors of the healthcare facility, 7 directors of nursing, 6 medical directors and 6 quality and risk management directors), 4 were middle managers (2 nurses and 2 physicians at the head of a division) and 13 were frontline managers (10 nursing managers and 3 physicians at the head of a care unit). Of the 44 managers, 25 (57%) reported having received management training. Seven top managers (6 directors, 1 director of nursing), 1 middle manager (nurse at the head of a division) and 6 frontline managers (nurse managers) participated in direct observations.

**Table 2:** Sociodemographic characteristics of participants (*n* = 65)

Participant characteristics	n	%
**Gender**		
Female	37	57
Male	28	43
**Age (years)**		
20–30	3	5
31–40	15	23
41–50	21	32
Over 51	25	38
No answer	1	2
**Staff position**		
Administration/ Executive management	27	41
Doctor/Surgeon	13	20
Nurse	18	28
Nursing assistant	7	11
Manager		
Yes	44	68
No	21	32
**Tenure with current facility (year)**		
Less than 1	3	5
1 to 5	8	12
6 to 10	18	28
11 or more	36	55
**Seniority in the current position (year)**		
Less than 1	2	3
1 to 5	15	23
6 to 10	26	40
11 or more	21	32
No answer	1	2
**Participation in a risk management entity**		
Yes	55	85
No	10	15
**Risk management training**		
During initial vocational training	7	11
During initial and continuing vocational training	6	9
During continuing vocational training	29	45
Don’t know	12	18
No answer	11	17
**Total**	**65**	**100**

### Representations of safety

**Fig. 1:**
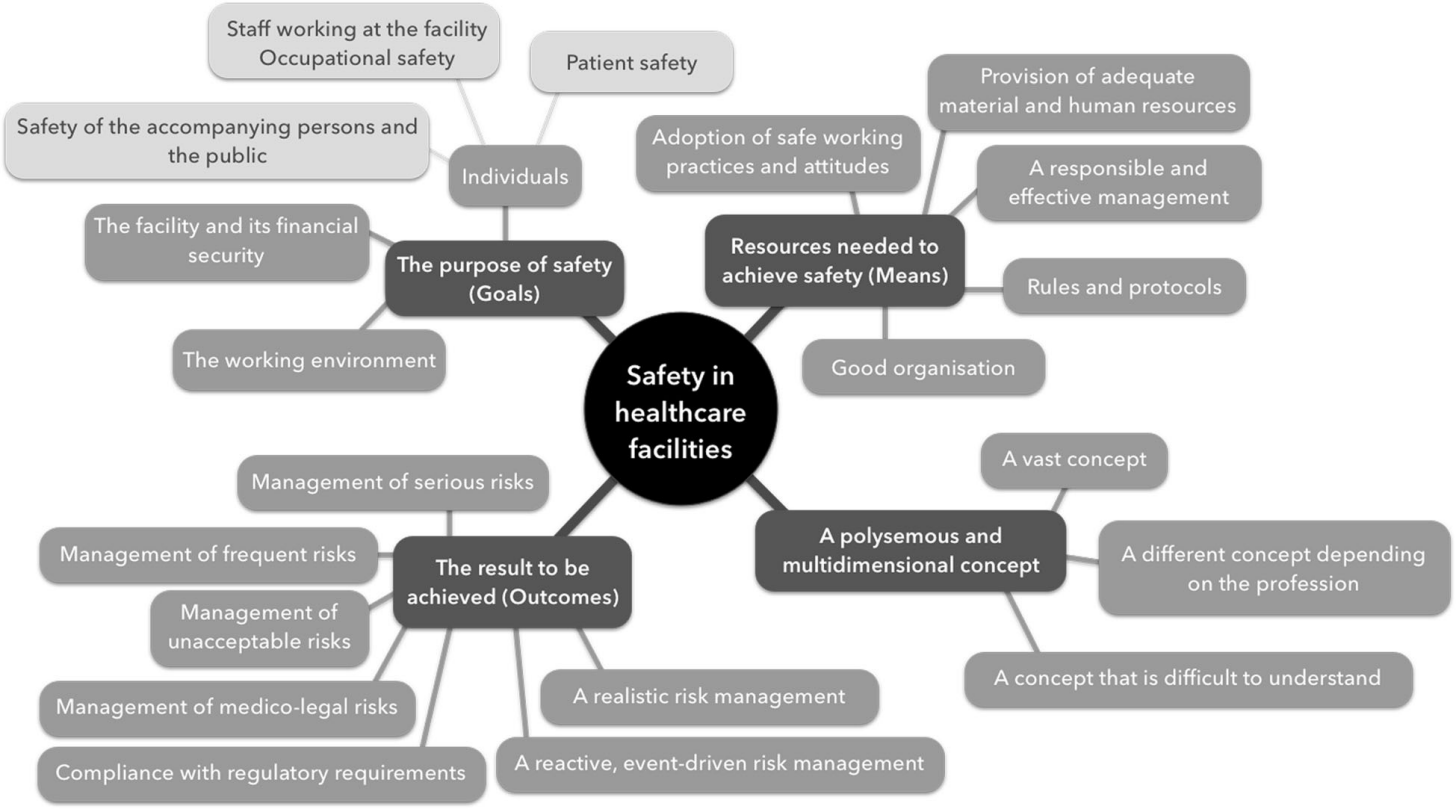
Themes used by participants (*n* = 65) to define safety in healthcare facilities

Although often defined as a vast and multidimensional concept, in this study we describe safety in healthcare facilities in terms of three main themes (Fig. 1): i) *the purpose of safety (goals); ii) the resources needed to achieve safety (means)*; and *iii) the result to be achieved (outcomes).* Examples of verbatim for each theme are provided in Additional file 2**.***The purpose of safety* includes individuals, the environment and the facility. The safety of individuals refers primarily to patient safety, but also occupational risks and the safety of accompanying persons and the public. Patient safety was perceived as a priority by both caregivers and managers, and was seen as not only linked to the risks associated with care, but also physical risks (i.e. self-injurious behaviour and aggressive behaviour directed towards other patients or staff) and environmental risks (i.e. fire). The priority given to patient safety is summarised in this statement from a nurse manager at facility E:

*“Really, the first objective is the safety of patient care, that is, that the patient has the care, treatment, examinations he needs and no more. He should leave in a better state of health compared to how he was when he came in.”*

Risks associated with care were mentioned more often in acute healthcare facilities, while physical risks were more of a concern in psychiatric facilities. Occupational risks were mainly mentioned by managers in the context of their negative consequences for patient safety, as expressed by a quality director (facility E):

*“What is interesting to consider when thinking about the safety of professionals is that occupational risks can also impact patients. This is what we see when teams are exhausted, in conflict or where there is a lot of absenteeism.”*

When safety was defined in terms of the resources needed to achieve it, the adoption of safe working practices was noted most often, primarily in terms of compliance with rules and protocols, followed by ethical practices considered to be inherent in any caregiving activity. A nursing assistant from facility B emphasized the importance of following good practice:

*“In terms of professional practices, I think that, in general, we have very good professional practices in the facility, and this contributes to safety.”*

The adoption of safe practices and attitudes also included knowing one’s own limitations and the non-technical skills that promote teamwork:

*“Knowing your own role, not going too far. Then it’s all about communication, teamwork” (Caregiver, facility A).*

The provision of adequate material and human resources, and effective management were two other issues that were regularly cited as a way to achieve safety, and were considered as managerial responsibilities:

*“You can't create a safety culture if you don’t already have a minimum level of environmental safety and high-performance equipment” (Quality Manager, facility A).*

When safety was defined as *the result to be achieved,* results were seen as either the management of the most serious or unacceptable risks (i.e. the death of a patient), the most frequent risks, or medicolegal risks. Professionals varied widely in how they saw results, for example: compliance with regulatory requirements; following a reactive, event-driven risk management approach; or the implementation a realistic risk management approach rather than the ideal but unachievable goal of zero adverse events.

Participants expressed positive views about the level of safety in their department and facility. This was mainly justified by the existence of a ‘shop-floor’ SC, in the form of competent professionals who generally followed good practice, had sufficient resources, and an institutional approach to improving safety. A nursing assistant from facility E stated:

*“We have good practices. We’re in a good department and the doctors are aware that they are in a department where we’re doing good work.”*

However, this shop-floor SC was also considered to be a work-in-progress, and sometimes even detrimental to safety, as expressed by a doctor (middle manager) from facility D:

*“There are always free electrons that are always difficult to control in this private context, especially with doctors. The institution can look away at times and, in my opinion, sometimes there’s not enough pressure and sometimes that suits us, too. But as a result, there are people who have bad practices, and no-one can do anything about it.”*

Support from frontline managers and sufficient resources, particularly material, were regularly cited as illustrations of positive perceptions of safety:

*When you need something, you tell the nurse manager [frontline manager] and she do everything she to make sure you get what you need. We don't have a problem with that* (Nursing assistant, facility A).

*“Our head of unit is very good in his role; he is very efficient! »* (Doctor, facility D).

*“If we are talking about equipment, yes, here, it’s all in the rules to work.”* (Nursing assistant, facility C).

On the other hand, lack of support from top managers and a lack of appropriate (notably human) resources to provide safe care were regularly mentioned as areas needing improvement, as expressed below in the quotes from three caregivers:

*“This summer, we weren't safe, we were short of staff. Every day there were staff on sick leave, sometimes having to be replaced at short notice, and all our director told us was that we had to get by. Afterwards, it wasn't the nursing manager's fault, he did everything he could to make up for the lack of staff.” (Nurse, facility F)*

*“We had a problem, risk of medication error for babies. I have proposed solutions and alerted our director to this recurring incident. (…) And I'm fighting to correct this problem, but an adverse event occurred (...) Nurses made that mistake of alerting the director and saying that we were not working well, not safe. But then again, since the last big problem, nothing has changed” (Doctor, facility D).*

*“one does not feel heard and supported by our direction. If only we would take the trouble to admit that things are not going well...”*

*(Nurse, facility C).*

This lack of support from the top management and its consequences was also mentioned by frontline managers, as expressed by this nurse manager from facility D:

*“Out of three weeks, I've had 9 caregivers in sick leave, and I didn't finish at 6:00 p.m.! Sometimes I didn't have a solution, sometimes I cracked up, I even cried because I didn't want to go home without finding a solution, but the top management does not provide a solution.”*

### Caregivers’ expectations of their managers and managerial perceptions of these expectations: a lack of coherence

There was only a partial overlap between caregivers’ expectations of their managers in terms of safety, and what managers perceived these expectations to be **(**Table 3**).** This gap between expressed and perceived expectations was found in each facility. Managers attributed more expectations to caregivers than the latter actually expressed. Conversely, two expectations expressed by caregivers were not perceived by managers: the allocation of time to talk about safety issues, and the promotion of a preventive risk management approach. A nurse from facility C expressed the need to take time to discuss about safety issues:

*“Currently, we’re trying to find a better way to manage external implants, (...) it's just getting going, but there’s still a lot of inertia (...) I'm hoping that my manager can find the time to talk about it, and that we can try, with the nurses and orderlies to look at this implant management, to try to find ways to improve its management.”*

A nurse from facility E, also expressed this need for creating work debate space around safety issues between managers and caregivers:

*“(...) I expect my nurse managers and director of nursing to find the moment to get together, and try with the nurses and nursing assistant who work around this issue, to try to find an improvement. ”*

On the other hand, managers perceived an expectation of reactive risk management approaches, such as the reporting and analysis of adverse events, an expectation not expressed by caregivers. Finally, one top manager (Quality Director) indicated that he did not know caregivers’ expectations of managers because he had no direct contact with them.

**Table 3:** Caregivers’ expectations of their managers (*n* = 21) and managers’ perceptions of these expectations (*n* = 44)

Category of staff	Safety expectations regarding managers
**Caregivers and managers**	▪ Provide satisfactory working conditions^a^
	▪ Enforce rules, check practices and correct deviations^a^
	▪ Organise health care
	▪ Acknowledge and value the work done by teams
	▪ Transmit information and promote training
	▪ Understand and spend time in the field
	▪ Listen and take into account the opinions of caregivers
	▪ Provide support in case of difficulty
**Only caregivers**	▪ Organise time to talk about safety issues
	▪ Encourage the implementation of preventive risk management approaches rather than reactive approaches
**Only managers**	▪ Promote adverse event reporting
	▪ Implement effective corrective actions
	▪ Involve caregivers in projects
	▪ Clearly define jobs
	▪ Communicate with senior management
	▪ Implement changes

^a^Expectation expressed by more than half of interviewees

Caregivers’ expectations fell into two main categories. The first was the provision of satisfactory working conditions, in particular, sufficient human resources to optimise the organisation of care and guarantee patient safety. A doctor from facility F indicated that:

*“it would be having enough resources to keep working, it’s trying to keep what you have. It’s not even asking for more, especially in terms of staff, but trying to keep what you have, and since the move is more towards reducing finances, it’s difficult...”*

This expectation was also perceived by managers, although they seemed more concerned with material than human resources:

*“Employees can have expectations regarding their working conditions and patients, if there are things that aren’t working, poor equipment” (Director, facility D).*

A second caregiver’s expectation was that managers should monitor their work. They expected managers to enforce rules, check practices and correct deviations:

*“You still have to remind people of the rules. I think that the role of the medical director is to refocus on the right rules. In terms of safety, we need to refocus. (...) Unfortunately, from time to time, you have to bark a little” (Doctor, facility F).*

This caregivers’ expectation that managers regulate their practices was perceived by managers, as a top manager pointed out:

*“When I'm familiar with the recommendations, I don’t hesitate to issue reminders. I do that on a regular basis”* (Medical Director, facility A).

Although this expectation was perceived by managers, the latter tended to consider that caregivers expected recognition, rather than regulation:

*“Staff expect recognition, and it’s true, in other words, not just telling them “it’s necessary”, “it’s the rule”, they still need to feel that they are valued and recognised. Knowing how to value them is very important, knowing how to acknowledge good work” (Director of Nursing, facility A).*

### Activities attributed to managers and actual managerial activities

The main activities attributed to managers by interviewees (44 managers and 21 caregivers), and observed safety-related activities (14 managers) are presented in Additional file 3 and Additional file 4 respectively.

Most of the activities attributed to managers by interviewees were also observed during direct, on-site observations. These activities, which were both attributed to managers and observed could be divided into six main categories: leading and motivating; monitoring and checking healthcare practices and organisation; communicating information and tools related to safety; coordinating the work of different departments; setting an example; and directing safety policy and its implementation. These six categories were homogenous across facilities, with the exception of facility G, where setting an example, communication and decision-making were less frequently cited than in other facilities.

However, observed activities differed as a function of the managerial level. We can take the example of monitoring and checking. For frontline managers, this mainly took the form of checks of caregivers’ professional practices. One example concerned a nurse manager who, while present on the ward, checked that porters had passed through the hospital’s reception before transferring a patient to the ward, and reminded them of the need to do so. As for top managers, a Director of nursing was observed reminding caregivers of the importance of following the facility’s identity monitoring procedure. Planning and keeping up to date were activities that were observed more frequently than they were stated, especially among frontline managers. Planning was mainly observed for frontline managers and concerned short-term tasks that mainly aimed at compensating for the absence of one or more caregivers. The nurse manager from facility G was observed managing the replacement of a nurse on sick leave, the director of nursing from facility D has been seen managing rotas for the department’s paramedical staff and midwives, taking into account holidays, time off for managers, sick leave, and unforeseen absences.

Finally, although rarely attributed to managers by interviewees, the presence of top managers in healthcare units was observed in two facilities (D and G). In facility D, the Director of nursing was observed meeting with a nurse manager and a technician repairing toilets in patient rooms. During his visit to the units, the director of facility G was observed mainly pointing out the shortcomings of caregivers in terms of hygiene and reminding the nurse managers that they should plan the implementation of corrective actions decided upon after the occurrence of adverse events.

## Discussion

This study showed that the professionals who were interviewed (44 managers and 21 caregivers) expressed positive perceptions of the level of safety in their facility. Support from frontline management was particularly appreciated, while support from top managers was identified as an area for improvement. However, caregivers’ expectations of their managers and managerial perceptions of these expectations only partially overlapped. Provide satisfactory working conditions, enforce rules/ check practices/ correct deviations, organise health care, acknowledge and value the work done by teams, transmit information and promote training, understand and spend time in the healthcare unit, listen and take into account the opinions of caregivers, and provide support in case of difficulty were expectations regularly perceived by both caregivers and managers. Organise time to talk about safety issues and encourage the implementation of preventive risk management approaches rather than reactive approaches were two expectations expressed by caregivers but not expressed by managers. Six main categories of safety-related activities were both observed among managers and regularly expressed by participants: leading and motivating; monitoring and checking healthcare practices and organisation; communicating information and tools related to safety; coordinating the work of different departments; setting an example; and directing safety policy and its implementation.

Our results showed that for healthcare professionals (managers and caregivers), the notion of safety within a healthcare facility was polysemic. While patient safety and its management were at the forefront, many other dimensions that seemed to be of concern to professionals were mentioned, such as their own safety and the financial safety of their facility, and mainly for their potential impacts on patient safety. The links between these different dimensions of safety and their impact on each other have been highlighted in recent studies, as the impact of professional safety and patient safety [33]. Furthermore, our results confirm that safety is a broad concept with multiple dimensions that are interrelated; and healthcare professional (managers and caregivers) have to deal with all these interrelated and often contradictory risks to achieve their duties with a satisfactory level of performance [34].

Several French surveys of the safety climate in healthcare facilities have found that “Managerial support for patient safety” is one of the least-developed dimensions [7, 21, 22]. Our qualitative study seems to confirm these earlier results and highlights that support and involvement from frontline managers seems to be perceived as more important than that of top managers. Our results are also consistent with Pronovost [24], at John Hopkins Hospital (Baltimore, Maryland, United States), who found that staff perceived greater support for patient safety was provided by their direct supervisors than senior leaders.

Our finding of a perception of insufficient support for patient safety from managers does not seem to have changed since earlier quantitative studies carried out in France [7, 21, 22]. Our results confirm those from a recent mixed-method study conducted in France that shown an association between, on the one hand, the patient safety culture dimensions scores, and on the other hand, the qualitative perception of SC by caregivers [35]. Particularly, this study found an association between the low score to the “Hospital management support for patient safety” and the negative perception of management’s support in open comments and interviews. This lack of progress is unlike studies carried out in the United States, in particular, facilities included in the Agency for Healthcare Research and Quality database, and measured with the same assessment tool [36]. The progress that has been observed in the United States could be linked to the implementation of actions aimed at strengthening the role of managers with regard to safety, in particular structured visits by different levels of the managerial hierarchy [37, 38].

In our study, the need to allocate time to discuss safety issues was clearly expressed by caregivers. However this expectation was not perceived by any of the participating managers, despite the importance of developing a shared vision of risks in fostering SC [18]. Based on the encouraging results obtained in the United States, the French National Health Authority (*Haute Autorité de Santé*) recently introduced an experimental safety walkaround in 13 pilot healthcare facilities, which is expected to be gradually rolled out to other institutions [39].

The current work of top managers and, particularly, healthcare facility directors is complicated by the fact that they are increasingly held responsible for results, good or bad. Moreover, they must ensure the smooth running of their facility, while the health system is under pressure as never before [40]. This, sometimes contradictory logic implies trade-offs between efficiency, legal considerations and resources, potentially leading to a failure to follow regulations and meet prescribed standards. Directors of large facilities tend to process and prioritise ‘files’, one of which is safety, as a function of the sensitivity of the topic and its perceived urgency.

Classically, two styles of leadership are distinguished [41]. The first (transactional style) is focused on short-term commitments that are based on explicit or implicit contractual relationships between the leader and his/ her followers. The aim is to achieve objectives, duties are clearly assigned and there is a system of sanctions and rewards. The second (transformational style) is characterized by the importance given to negotiation in the definition of each follower’s tasks. Here, the aim is to achieve objectives through developing the intrinsic motivation of followers, and a sense of shared mutual interests.

In most of the healthcare facilities that participated in our study, managers had implemented a style based on transformational leadership [41]. They were trying to build a SC based on motivation, communication, individual commitment and consideration for individuals. While caregivers were sensitive to this style, they also expressed high expectations with respect to transactional leadership. In particular, they expected their managers to monitor practices and the organisation of care, clearly define their expectations, objectives and priorities, and establish roles and objectives for each staff member.

Another important finding of our study is that although caregivers wanted to see the development of a more dynamic approach, in the form of prevention initiatives based on a proactive view of safety, managers tended to favour reactive, risk management approaches such as adverse event analyses. This result highlights the need to train managers in the most up-to-date safety management models, which place greater emphasis on proactive, rather than reactive, risk management methods [42]. In this context, the recent safety management approach developed in the field of organisational resilience engineering, is particularly relevant [43]. This perspective no longer considers safety as a reactive approach focused on avoiding adverse events (the traditional perspective, named “Safety-I”). Instead, safety is seen as the capacity to succeed, in other words, the ability to provide adequate care to patients under ever-changing and variable working conditions (the so-called “Safety-II” perspective). Our study found that while managers had a vision of safety that was more consistent with the Safety-I perspective, caregivers, who are directly exposed to patients, tended to expect the implementation of approaches based on a Safety-II perspective. One example of this was their desire to see time set aside to talk about safety.

Our study also highlighted different categories of activities that foster safety, and gaps between caregivers’ expectations of managerial support for patient safety and managerial perceptions of these expectations. Such gaps between expressed and perceived expectations seem to be as important as cultural gaps between caregivers and managers [44].

Our study has various methodological limitations. Notably, there are several possible biases: selection – linked to the fact that facilities volunteered to participate; social desirability [45] – linked to the interviewee; and observation – during the monitoring of a manager’s day. However, the methodology followed several of Parand’s [29] recommendations, which made it possible to explore the components (focused on SC) of the input-process-output management model. This model is used to conceptualise the factors that contribute (input) to managerial activities (process) that impact on quality and safety (output). In particular, we studied the role of managers in developing the quality of care and patient safety. We examined the activities of all managerial levels – not only top managers but also middle and frontline managers. We also looked at the perceptions of non-managers; we analysed the time spent and tasks performed by top managers beyond their participation on the board of directors; and we studied organisational and individual factors of managers.

### Implications / recommendations

Our findings support the need to allocate more time for managers and caregivers to discuss safety issues, for instance in the form of a safety walkaround [38, 39, 46] or other structured work debate space as implemented in other industries [47]. This research created an opportunity for the seven participating healthcare facilities to conduct an analysis of their governance and organisation of risk management, and to draw up recommendations aimed at developing a SC. An extract from one of these recommendations is provided in Table 4**.**

**Table 4:** Extract from the report sent to one facility (Facility D)

“Several results of the study can explain the potential for improving safety culture: an inconsistent vision of safety that is not shared, especially between caregivers; the perception of a good level of safety in the facility by professionals noted in interviews which may lead to a reduced commitment to safety; various expectations expressed by caregivers toward their managers but not perceived by these latter (standardise the rules for patient admission, computerisation of handover and patient fields, more regulation of practices and guidance in case of conflict between patient safety and productivity, listening and attention). Both caregivers and managers perceived the need to have good working conditions. Better communication between managers and caregivers, and understanding caregivers’ expectations, could be useful in developing a safety culture.”	

## Conclusions

The present study confirms that managers have a crucial role to ensure the development of a SC in healthcare institutions. It highlighted that support from, and the involvement of frontline managers seems to be perceived as more important than that of top managers. This study also identified categories of activities that foster safety, and highlighted some gaps between caregivers’ expectations of their managers and managerial perceptions of these expectations. This result could be used to develop training programs to help healthcare managers understand their crucial role in the development of SC. Our findings also support the need to allocate more time for managers and caregivers to discuss safety issues, such as safety walkaround [38, 39, 46] or structured work space debate on actual practices [47].

## Supplementary information

**Additional file 1: **Semi-structured interviews guide used with managers (*n* = 44) and caregivers (*n* = 21).

**Additional file 2: **Example of verbatim for each themes and sub-themes regularly cited by participants (*n* = 65) to define safety and principal risks in healthcare facilities.

**Additional file 3: **Main categories of activities intended to improve safety attributed to managers by managers (*n* = 65) and caregivers (*n* = 21).

**Additional file 4: **Categories of safety-related activities observed among managers (*n* = 14).

## Data Availability

Datasets generated and/or analysed during the current study are available upon request from the corresponding author, JLQ, at jean-luc.quenon@ccecqa.asso.fr

## Abbreviation

SC: Safety culture
