# Supplementary material for: Exploring the role of managers in the development of a safety culture in seven French healthcare facilities: a qualitative study
Source: BMC Health Serv Res. 2020 Jun 8;20:517. doi: 10.1186/s12913-020-05331-1 (PMC7278117; doi:10.1186/s12913-020-05331-1)
Supplement: Supplementary file 1 — Additional file 1: Semi-structured interviews guide used with managers (n = 44) and caregivers (n = 21). [file 12913_2020_5331_MOESM1_ESM.docx]

**Additional File 1.** Semi-structured interviews guide used with managers (n = 44) and caregivers (n = 21).

**At the beginning of the interview - all participants**

I’m [first name and last name of the interviewer], a researcher from the “Comité de Coordination de l’Evaluation Clinique et de la Qualité en Nouvelle Aquitaine”.

First of all, I would like to thank you for agreeing to receive me today at your workplace for this interview. I would like to question you for our study. This study is financed by a research fund of the Ministry of Health. Its objectives are to explore caregivers and managers’ perception of safety in their healthcare facility, the role of their managers in the development of safety culture and the managers’ activities related to the development of safety culture in their healthcare facility.

In order to allow better exchanges and to be able to be as accurate as possible with your answers when I will write them down, I would like to record the interview with this audio-recorder. Do you give me permission to record this interview?

**If, yes**, ACTIVATE THE RECORDER AND CONTINUE

**If, no** => STOP

This study based is based on a one-hour semi-structured interview and a one-day direct on-site observation. It does not expose you to serious physical, psychological or social risks. For you, participation in this study implies answering the questions I would like to ask you during this interview, which is expected to last about one hour. This interview is confidential and completely voluntary. You have the right to refuse to participate in the study or to withdraw consent to participate at any time without having to justify yourself to anyone or provide an explanation. If we should come to any question that you do not want to answer, just let me know and we will go on the next question. If you would like to take a break during the interview, let me know and I will stop the recorder and we will resume the interview when you decide to do so. At the end of the study, we will organize a collective feedback session on its overall results. If you wish to receive these results directly, just send me a message to the e-mail address in the information leaflet.

Have you understood what is being asked of you?

If so, do you agree to participate in this study?

**If, yes**, CONTINUE

**If, no** => STOP

**Questions for managers**

| **Domain** | **Questions** |
| --- | --- |
| Socio-demographic data | - Age - Sex - Profession / Current position - Seniority in the facility - Seniority in current position - Participation in an institutional safety body or a working group |
| Professional data | - What is your professional background? - Did your training include elements related to safety? - During your managerial training, have you been taught about safety in healthcare facilities? - What are your current sources of knowledge about safety? |
| Perception of safety | - What does safety mean to you in your healthcare facility? - At your level, what are the risks that you consider the most important to control? - How would you rate the level of safety in your facility: (excellent/ very good/ acceptable/ weak/ unacceptable)? - What do you base this on? |
| Safety actions | *Actions at the facility level*   - What is your role in setting the procedures and rules of the safety policy of your facility? - What is your role in developing safety in the safety policy of your facility? - What are the actions you take in relation to safety? - What changes in safety have you made in the last 12 months? What have been their effects?   *Actions at the division/department/unit level (middle & frontline managers only)*   - Who do you ask to develop safety actions at your level (division or department)? - What place do you give to the expression of caregivers in terms of safety? - Can you tell me about the last adverse event that occurred in your facility, in your division or unit or service; how did you manage it? - Do you report adverse event yourself, internally? What types of adverse event do you report? - Is this your usual way of doing things? - Is this way of doing things shared by other managers? |
| Support from hierarchy | - Do you value compliance with safety rules and caregivers' initiatives in term of safety? - Do your managers usually value your work? - Do you encourage professionals to report adverse event? - Do you encourage professionals to address both the immediate and underlying causes of adverse event? - How do you manage recurrent safety issues? |
| Safety expectation | - In your opinion, what are the safety expectations of your managers? - In your opinion, what do caregivers expect from managers in terms of safety? - How do you integrate these expectations into your work as a manager? - From your point of view, what do you think could be improved in terms of safety in your facility? |

**Questions for caregivers**

| **Domain** | **Questions** |
| --- | --- |
| Socio-demographic data | - Age - Sex - Profession (nurses, care assistants, doctors) - Current position - Seniority in the facility - Seniority in current position - Participation in an institutional safety body or a working group |
| Professional data | - What is your professional background? - Did your training include elements related to safety? - What are your current sources of knowledge about safety? |
| Perception of safety | - What does safety mean to you in your healthcare facility? - At your level, what are the risks that you consider the most important to control? - How would you rate the level of safety in your facility: excellent/ very good/ acceptable/ weak/ unacceptable? - What do you base this on? - Is this perception shared in the service? Could you tell me by whom? - What changes in safety has been made in the last 12 months in your unit? What have been their effects? - What do you expect from your managers in term of safety? |
| Safety actions | *Actions at the facility level*   - What is the role of your managers in setting the procedures and rules of the safety policy of your facility? - What is the role of your managers in developing safety rules and procedures in the safety policy of your facility? - What are the actions, the role of your managers in relation to safety?   *Actions at the division/department/unit level (middle & frontline managers only)*   - What role do your managers give to you and your team in the development of safe care practices and procedures in your department/unit? - Can you tell me about the last adverse event that occurred in your facility, in your department/unit; how your managers handle it? - Is the usual way of doing things? - Is it shared by other managers? |
| Support from hierarchy | - Do your managers encourage you to comply with safety rules? - Do your managers encourage you to report adverse event? - Do your managers encourage you to address both the immediate and underlying causes of adverse event? - How do your managers deal with recurrent safety issues? |
| Safety expectation | - In your opinion, what are the safety expectations of your managers? - In your opinion, what do think your managers expect in term of safety? - How do you integrate these expectations into your work as a caregiver? - From your point of view, what do you think could be improved in terms of safety in your facility? |

**End of the interview**

- Thank the participant for the time spent on the study.
- Inform the participant that all data collected will be treated confidentially and ask him/her again if he or she agrees to have the recorded data transcribed in full and analysed as part of this research project.
- Inform him/her that a collective feedback session will be organized at the end of the project, but if he/she wishes to obtain the results directly, he/she can ask us to do so either now or by e-mail.
