# Supplementary material for: Exploring the role of managers in the development of a safety culture in seven French healthcare facilities: a qualitative study
Source: BMC Health Serv Res. 2020 Jun 8;20:517. doi: 10.1186/s12913-020-05331-1 (PMC7278117; doi:10.1186/s12913-020-05331-1)
Supplement: Supplementary file 2 — Additional file 2: Example of verbatim for each themes and sub-themes regularly cited by participants (n = 65) to define safety and principal risks in healthcare facilities. [file 12913_2020_5331_MOESM2_ESM.docx]

**ADDITIONAL FILES**

**Additional file 2.** Example of verbatim for each themes and sub-themes regularly cited by participants (n = 65) to define safety and principal risks in healthcare facilities

| Themes^1^ | Sub-themes | Examples of verbatim |
| --- | --- | --- |
| The purpose of safety | People / patients | *“The first objective is the safety of patient care, that is, that the patient has the care, treatment, examinations he needs and no more. He should leave in a better state of health compared to how he was when he came in”* (Healthcare manager, facility E)  *“Avoid the death of the patient or rather no serious complications”* (Doctor, facility D)  *“The ultimate risk that comes to my mind is a patient who does something to another person. This is the riskiest aspect of the care we provide. Then it’s whether they hurt themselves. That’s what happens most often.”* (Doctor, facility F)  *“The most serious thing is the death of the person, if there’s a fire...”* (Director, facility G)  *“What concerns me the most is the safety of care because I’m not responsible for fire safety”* (Director of Nursing, facility E) |
|  | People / staff working at the facility | *“It’s well-being at work, feeling heard, or rather listened to, being able to talk to the hierarchy. Then there is everything that is safe in the sense that we shouldn’t put ourselves in danger either.”* (Nursing assistant, facility B).  *“It can also be the safety of caregivers, the working environment can be dangerous, especially in psychiatry. It can be the mental and emotional safety of caregivers.”* (Healthcare manager, facility F)  *“When handling cytotoxic products, I should wear (...) a mask and glasses but we rarely take the time to put all this on.”* (Nurse, facility E)  *“The most difficult to manage are all the psychological aspects, well, I mean any caregiver can burn out, especially in psych (...) for a long time I’ve been responsible for new recruits and students (...) the first thing I teach them is to protect themselves by putting themselves inside a bubble. ‘You put on the uniform, you put yourself inside the bubble’.”* (Healthcare manager, facility G)  *“Occupational risks can also impact patients. This is what we see when teams are unhappy, in conflict or where there is a lot of absenteeism.”* (Quality director, facility E) |
|  | People / accompanying persons and the public | *“To reduce risks as far as possible for all of the people who have to pass through the facility, whether patients, caregivers or accompanying persons”* (Doctor, facility C)  *“Our first mission, as a public facility, is to ensure the safety and security of our premises, our professional environment and provide a safe environment for the public”* (Quality director, facility D) |
|  | The working environment | *“(...) do everything possible to ensure that there is a general level of safety: electrical safety, safe operation of operating theatres, with filtered air, (...) electricity, water...”* (Doctor, facility C)  *“Then there is environmental safety (...) there is safety at the building level, finally at the premises level, which is more the responsibility of the technical team who we are permanently linked to anyway. "* (Healthcare manager, facility A) |
|  | The facility and its financial security | *“Then there is the financial risk, which can also have an impact on patient safety. If, for example, we want the best for them, it’s obvious that we’ll have to save money elsewhere. If we want to shareholders to receive dividends, we cut back on the quality of certain things...”* (Quality Director, facility C)  *“Three years ago, we almost made a loss, I can tell you that the only thing we would have talked about then would have been the balance sheet*.*”* (Top manager, facility D) |
| Resources needed to achieve safety | Attitudes and practices of professionals | *“We, if we assess that there is a significant risk [of suicide], we put a protocol in place (...); we leave the patient in their pyjamas for 24 or 12 hours (...). I’m not a freedom fighter, but well, I’m here for patient safety”* (Doctor, facility G)  *“But after that it’s everyone, all the doctors, as soon as there’s a protocol in place, we stick to the protocol and follow the protocol”* (Head of Unit, facility A)  *“We’re in a good department and the doctors are aware that they’re in a department where we work well together. If they weren’t happy, we’d know about it!”* (Nursing assistant, facility F)  *“I’ll explain it to you as a metaphor. In business, there are three criteria for things to work well – location, location and location. And in medicine, there are three criteria: rigour, rigour and rigour.* (Medical director, facility C)  *“Safety is also about being there if something happens all of a sudden (...) you always have one eye looking behind you.”* (Nursing assistant, facility G)  *“It’s up to us to make sure that even if there are two of us, we still have to ensure that the department is safe. Which is why we also work as a team.”* (Nursing assistant, facility G)  *“Knowing your own role, not going too far. Then it’s all about communication, teamwork.”* (Caregiver, facility A) |
|  | Suitable human and material resources | *“You can't create a safety culture if you don’t already have a minimum level of environmental safety.”* (Quality manager, facility A)  *“In terms of equipment, I think we are lucky to be in an facility where we are well equipped, I would like to point this out, because I think it is... not everyone works in a place where the equipment is such good quality.”* (Healthcare manager, facility B)  *“(...) it requires high-performance equipment and a team with advanced medical skills. And people who provide this safety. (...) We need reliable people.”* (Director, facility B)  *“Depending on whether you decide to put an additional nurse position here or there, (...) depending on whether you decide to buy a particular type of medical equipment or not, there may be underlying safety issues! If we don’t buy this equipment, if we make that choice, we can remove an opportunity for the patient who would have needed that examination.”* (Director, facility E) |
|  | Responsible management | *“(...) in some way, I think that it’s really part of the manager’s job to manage quality and safety (...) We’re still responsible for the safety and organisation of care in the unit we are in charge of.”* (Healthcare manager, facility A)  *“A manager who does not support his team or who isn’t present will create insecurity because the team feels abandoned (...) teams need a leader who understands them, who listens to them, otherwise negative phenomena appear and power relations develop in units that the manager is unaware of.”* (Director of Nursing, facility F)  *“If there is a safety breach, be it infrastructure, generators for example (...) that is the direct responsibility of senior managers.”* (Doctor, facility B) |
|  | Rules and protocols | *“We already have protocols that have been put in place for the proper functioning of a department, for patient safety and for our safety as well.”* (Nursing assistant, facility F)  *“[Safety] is about protocols and processes that everyone knows.”* (Medical director, facility E) |
|  | Good organization | *“It’s also the facility of consultative bodies (...) to facilitate and improve the safety of care.”* (Healthcare manager, facility A)  *“It’s a question of organization where each link in the patient care chain has an impact on the others.”* (Director of Nursing, facility B) |
|  | Appropriate systems | *“Let's say that for me, safety is something that principally concerns the patient, and what we have to offer him in terms of guarantees in terms of admission and possible follow-up at discharge. In practice, everything that is done by the different actors must contribute, for me it’s at this level.”* (Director of Nursing, facility F) |
|  | | |
| The result  to be achieved | Management of serious risks | *“As for patients, if you make a mistake when you’re treating them, it can be serious, it can be fatal. So that’s what is most important.”* (Nurse, facility A) |
|  | Management of frequent risks | *“The ultimate risk that comes to my mind is a patient who does something to another person. In our care, this is the riskiest aspect. Then it’s whether they hurt themselves. That’s what happens most often.”* (Doctor, facility F) |
|  | Management of unacceptable risks | *“Because there are already enough studies that enable safeguards to be put in place on the wards and even though we are doing what’s needed, we still have problems and I don’t think that it is acceptable.”* (Director of Nursing, facility C) |
|  | Management of medico-legal risks | *“What they expect most and which is very surprising is legal safety, in relation to what they do: ‘Do we have the right to do that?’. They expect a lot from management in this respect and I’ll give just one striking example: in intensive care, I’ve been asked several times to advise on very delicate end-of-life cases and in these cases, doctors and caregivers expect a lot from management in terms of setting out the legal framework and keeping up to date.”* (Quality director, facility E) |
|  | Compliance with regulatory requirements | *“You see, we still have a bias in our approach, because in the end we are obliged to rely on this certification visit and to prioritize actions because we will have to answer for them.”* (Quality director, facility D) |
|  | Reactive, event-driven risk management | *“Often our day-to-day life makes the news”* (Top manager, facility D)  *“It’s events that drive us to address this or that safety problem.”* (Director, facility C) |
|  | Realistic risk management | *“There is no such thing as zero risk”* (Director, facility E) |
|  | | |
| A concept that is vast, multidimensional and difficult to define | A vast and multidimensional concept | *“For all problems, including those that do not appear to be related to safety, there is clearly a safety dimension, whether related to care or related to the organization.”* (Director, facility E)  *“[Safety is] an extremely broad theme, because safety is in so many areas.”* (Director of Nursing, facility D) |
|  | A different concept depending on the profession | *“It’s so vast! It depends on your profession.”* (Nursing assistant, facility C) |
|  | A concept that is difficult to understand | *“We can talk about it for days and we can also only talk about what we want to.”* (Medical director, facility D)  *“It’s so multifactorial in a healthcare facility that it’s quite difficult to come up with a single definition of safety.”* (Director, facility E) |

^1^ A theme was considered to be regularly cited if it was expressed by at least two participants.
