# Supplementary material for: Exploring the role of managers in the development of a safety culture in seven French healthcare facilities: a qualitative study
Source: BMC Health Serv Res. 2020 Jun 8;20:517. doi: 10.1186/s12913-020-05331-1 (PMC7278117; doi:10.1186/s12913-020-05331-1)
Supplement: Supplementary file 3 — Additional file 3: Main categories of activities intended to improve safety attributed to managers by managers (n = 65) and caregivers (n = 21). [file 12913_2020_5331_MOESM3_ESM.docx]

**Additional file 3:** Main categories of activities intended to improve safety attributed to managers by participants (n = 65).

| Category of managerial activity |  |  | | Main managerial activities^1^ attributed to managers | | | | | |  |  |  |
| --- | --- | --- | --- | --- | --- | --- | --- | --- | --- | --- | --- | --- |
|  |  | Managerial activity | | | | |  | Examples of verbatim |  |  |  |  |
| Lead and motivate |  | - Encourage staff to express themselves, in particular by considering their problems and suggestions - Motivate staff by encouraging them to become involved in risk management or participate in safety-related projects - Promote individual respect for good practice through incentives, most often oral - Acknowledge the work of teams when they have succeeded | | | | |  | *“(...) I think you need a little bit of time when you are available to teams (...) which is not obvious on the day-to-day level. And that they feel that you’re available, ready to talk, anyway.”* (Healthcare manager, facility A)  *“We’re engaged in all possible processes although there is a lot of discouragement sometimes (...) but we mustn’t get discouraged and do what we have to do. I’m here to get things moving again (...) Keep us on track.”* (Director, facility B) |  |  |  |  |
| Monitor and check the practice and organization of care |  | - Evaluate professional practices and skills - Monitor the organization of care with a view to improvement - Remind staff of good practice and their professional responsibilities (on a daily basis, when integrating new staff or changing the number of staff or beds) - Sanction professional deviance, especially when it involves repeated or deliberate behaviour - Check that regulations issued by supervisory bodies are being followed | | | | |  | *“Regarding drugs, it’s simple, yesterday morning, I opened the drug cart and checked if it was labelled, if it had the right name. Then, if it’s not labelled, I do it right away... (...) I do it but then I go to the person and tell them that they haven’t done it.”* (Healthcare manager, facility E)  *“They haven’t yet adopted a prevention culture, above all they* [supervisory bodies] *have a sanction culture. I’m the lightning rod for teams.”* (Director, facility F)  *“You still have to remind people of the rules. I think that the role of the medical director is to refocus on the right rules. In terms of safety, we need to refocus. (...) Unfortunately, from time to time, you have to bark a little”* (Doctor, facility F). | | | |  |
| Communicate information and tools related to patient safety |  | - Provide professionals with the information and tools they need to ensure patient safety - Provide feedback to professionals on the results of evaluations - Feedback safety information to senior management | | | | |  | *“A director’s job often involves rehearsing. There are 1500 people to convince, I don’t see them all, they don’t all know me, so I have to find a way to present my ideas to different bodies, to the teams when I meet them.”* (Director, facility E)  *“You have to explain things to teams. So, we are lucky to have, as I told you, a monthly staff meeting where we can talk to teams with a cool head, away from patients, where we can say things and report back on some of the actions we have taken.”* (Medical director, facility D) | | | |  |
| Coordinate professionals and safety actions |  | | - Collaborate with other managers (mainly between top managers and between top managers and frontline managers) - Collaborate with healthcare professionals working in their department(s) - Collaborate and facilitate coordination with professionals in other departments (pharmacy, accounts), risk management bodies, the executive secretariat, patients, partners, unions, etc. - Coordinate the implementation of risk management - Coordinate risk management bodies and provide secretarial support - Act as an intermediary between caregivers and top managers | | |  | | *“The idea is still to work with the managers and feed information up and down.”* (Director of Nursing, facility F)  *“Every Thursday, we have a one-hour meeting between doctors and administrative staff (...) it’s a time for doctors, the director and administrative staff to come together. And there’s a whole pile of things that are discussed, that are in progress, and decisions are made, it’s like a mini institutional medical conference every Thursday and that’s fantastic.”* (Doctor, facility G). | | | | |
| Set an example |  | | - Participate in consultative bodies and working groups - Provide a substitute or alternative, be available during rest periods - Apply safety rules - Challenge practices - Be responsible for safety (intermediary, point of contact) - Participate in safety-related projects - Ensure that safety budgets are maintained | |  | | | *“(...) it’s a bit up to me to set an example. Working together, all these projects, rigour, seriousness, it’s still the head of the division who must show the way. We must look at everything that’s said to us, even if it’s painful.”* (Head of division, facility A)  *“Currently, (...) I replace the medical secretary from 4:30 pm (...). Sometimes, I replace nurses when they are short staffed. I’ve also had to (...) replace a nursing assistant.”* (Healthcare manager, facility G). | | | | |
| Direct safety policy and its implementation |  | - Define values and objectives for the facility - Make trade-offs and validate, usually by working with others, the multitude of decisions taken independently by various managers (like the conductor of an orchestra) - Allocate resources (define staffing levels, the purchase of care systems, manage budgets) | | | | |  | *“It’s not the role of the facility’s manager to define precisely what the risk management policy looks like, there are so many options but, I would say, it’s not up to him to describe everything in detail, otherwise he would have to be a specialist in risk management, human resources, finance, medicine, biomedicine, procurement, it’s impossible~~.~~ (...) After that, either together, or just the facility’s director working alone, we decide whether or not to go, whether or not to push, whether to slow down or go faster...”* (Director, facility D)  *“Sometimes, it’s complex. I quite often find that I have to decide between the safety requirements of different professions.”* (Director, facility E). | | | |  |
| ^1^A category of managerial activity was considered as mainly attributed to the manager when it was attributed by more than half of participants. | | | | | | | | | | |  |  |
