# Supplementary material for: Exploring the role of managers in the development of a safety culture in seven French healthcare facilities: a qualitative study
Source: BMC Health Serv Res. 2020 Jun 8;20:517. doi: 10.1186/s12913-020-05331-1 (PMC7278117; doi:10.1186/s12913-020-05331-1)
Supplement: Supplementary file 4 — Additional file 4: Categories of safety-related activities observed among managers (n = 14). [file 12913_2020_5331_MOESM4_ESM.docx]

**Additional file 4:** Categories of activities safety-related activities observed among managers (n = 14)

| Category of managerial activity |  | |  |  | Managerial activities observed | | | | |  |  |  |
| --- | --- | --- | --- | --- | --- | --- | --- | --- | --- | --- | --- | --- |
|  |  | | Managerial activity | | |  | Examples of observed safety-related activities | |  |  |  |  |
| Lead and motivate |  | | Encourage staff to express themselves and consider their problems and suggestions  Motivate staff by encouraging them to become involved in risk management or participate in safety-related projects  Promote individual respect for good practice through incentives, most often oral  Value the work done. Acknowledge the work of teams when they have succeeded | | |  | Listen to the difficulties and complaints of caregivers” (Director of Nursing and Nurse manager, facility A).  Respond to a request for an appointment from a nurse who wished to discuss a problem (Nurse manager, facility B)  Take into account caregivers’ points of view regarding the unit’s staff rotas (Nurse manager, facility D).  Free up a slot in the schedule to talk to a healthcare manager about his/ her difficulties in organising care and staff rotas following repeated sick leave absences due to exhaustion (Director of Nursing, F).  Ask Nurse managers to join in consultations on a new way to organise staff rotas (Director of Nursing, B).  “Gather opinions from the department’s caregivers regarding the implementation of a new system for storing equipment (Nurse manager, facility B)  Encourage two nurses to participate in the evaluation of two boxes of medical devices used in the department in order to determine whether it was appropriate to standardise the equipment used (Nurse manager, facility C).  In the context of the forthcoming restructuring of a department, encourage middle managers to participate in meetings aimed at defining the minimum number of staff required to provide safe care and ensure the continuity of work (Director General, facility D).  Motivate a nurse to prepare posters to inform patients about safe care (Director, facility G).  Encourage a doctor to visit a patient (Director of Nursing, facility A)  Express satisfaction with the work done by a student nurse and the supervision provided by his tutor during an internship that was coming to an end (Nurse manager, facility D). | |  |  |  |  |
| Monitor and check the practice and organization of care |  | | Evaluate professional practices  Monitor the organization of care with a view to improvement  Remind staff of good practice and their professional responsibilities (on a daily basis, when integrating new staff or changing the number of staff or beds)  Sanction professional deviance, especially when it involves repeated or deliberate behaviour  Check that regulations issued by supervisory bodies are being followed | | |  | During a meeting, remind nurse managers of the procedure for managing medicines and for nursing staff to identify patients (Director of Nursing, A).  Check the management of the day’s meal orders, in accordance with the facility’s procedures (Nurse manager, facility A).  During a meeting, ask nurse managers to remind teams of the rules for organising breaks (Director of Nursing, facility B).  Remind departmental staff that they must not forget to provide all patients with a satisfaction questionnaire (Nurse manager, facility B).  Check stocks of a product used by the department (Nurse manager, facility B).  Make an unexpected change to the schedule for an operating theatre, in response to the failure of a piece of equipment (Nurse manager, facility C).  During an endoscopy examination, remind the medical and paramedical team of hygiene rules regarding soiled equipment (cover used equipment with a surgical drape) (Nurse manager, facility C).  During a meeting, remind Nurse managers of their role in the certification of the facility (Director of Nursing, facility C).  Welcome a new healthcare professional to the ward, remind him/ her of the dressing procedure before entering the operating theatre and check that it is correctly applied. (Director of nursing, facility C)  Remind staff new to the department of good practices and set out their responsibilities (Director of Nursing, facility D).  Correct inappropriate practices. For example, ask paramedics if they had notified the patient admission’s office of a new patient and remind them to have the patient admitted before transferring him/ her to the department (Nurse manager, facility D).  Respond to changes in the organisation of care, i.e. modify rotas to compensate for staff absences (Nurse manager, facility D).  During a Medical Committee meeting, read out the regulatory texts relating to the obligations of professionals with respect to on-call work (Director General, facility E).  Following a complaint from the trade union, remind Nurse managers that it is forbidden to record staff who are on strike in departmental rotas (Director of Nursing, facility F).  Report violations of hygiene rules after a visit to a department (Director, facility G).  Remind Nurse managers that they must implement planned corrective actions following an adverse event (Director, facility G).  During the weekly medico-administrative staff meeting, remind doctors of regulations governing the sending of patient discharge letters (Nurse manager, facility G).  Carry out safety visits. Check the hygiene of premises and resolve difficulties identified by teams (Director, facility G). | | | | |  |
| Communicate information and tools related to patient safety |  | | Provide professionals with the information and tools they need to ensure patient safety  Provide feedback to professionals on the results of evaluations  Give feedback safety information to top-management | | |  | During a meeting, present organisational changes, in particular, the work of Nurse managers will be divided up as a function of their position (Director of Nursing, facility B).  Run ‘flash’ meetings to make nurses, care assistants and maintenance workers aware of new arrangements for the facility’s Nurse managers (Nurse manager, facility B).  Put up the rota and a new procedure in the staffroom (Nurse manager, facility D).  Put up a poster in the emergency department that reminds staff of the importance of securing the medication circuit and checking the identity of patients, as part of National Patient Safety Week organised within healthcare institutions (Director of Nursing, C).  Communicate information about patient care and management within the department (Nurse manager, D).  Inform staff about new organisational arrangements (report on the worrying financial situation of the facility (Director General); report on the results of a certification visit (Director General, E).  Contact the quality director regarding repeated breaches of hygiene rules by certain operating theatre personnel, i.e., leaving the operating theatre wearing scrubs, seek a solution to this hygiene problem (Director of Nursing, D).  Alert management to safety problems associated with the implementation of a new patient identification procedure within the facility (Head of Unit, E).  Explain to Nurse managers how to run an internal audit to evaluate the management of pain, scabies, influenza and tuberculosis within their departments (Director of Nursing, F).  Encourage Nurse managers to report difficulties encountered by personnel who use protection devices for isolated workers (Director of Nursing, facility F).  Inform senior management of absences in the department and consequences for the workload of remaining staff (Nurse manager, facility F).  Transmit patient information to healthcare professionals working in the department (Nurse manager, facility F).  Inform a doctor about changes to how the department is organised, and new working hours (Director, G).  Present new working practices to doctors (Director, facility G).  Read adverse event report cards that the facility’s Director had asked to be reported during the weekly medico-administrative staff meeting (Nurse manager, facility G). | | | | |  |
| Coordinate professionals and safety actions |  | Collaborate with other managers (mainly between top managers and between top managers and frontline managers)  Collaborate with healthcare professionals working in their department(s)  Collaborate and facilitate coordination with professionals in other departments (pharmacy, accounts), risk management bodies, the executive secretariat, patients, partners, unions, etc.  Coordinate the implementation of risk management  Coordinate risk management bodies and provide secretarial support  Act as an intermediary between caregivers and top managers | | | |  | | Work with healthcare staff in the department to manage patient admissions and discharges (Nurse manager, facility A).  Discuss patient care with other departments (social services, mobile psychology teams, pharmacy, etc.) (Nurse manager, facility A).  Agree with the pharmacist on how to dispense a drug (Director of Nursing, B).  Contact the pharmacy to collect a drug and ensure the traceability of its delivery (Nurse manager, facility B).  Contact the delivery room by phone to notify professionals of a change in the room number assigned to a patient and her newborn (Nurse manager, facility D).  Contact the operating theatre to tell them that a child who is about to undergo surgery will only be accompanied by his father and not both parents as planned (Nurse manager, facility D).  Meet a Nurse manager who had just started sick leave following a fall in the facility, and discuss different options for his/ her replacement (Director of Nursing, facility D).  Inform a midwife of the dates of a patient’s blood transfusion and ensure that s/ he is able to trace the information (Nurse manager, facility D).  Collaborate with all of the department’s personnel (childcare assistants, nurses, midwives, doctors) on the management of several patients (Nurse manager, facility D).  Discuss the hospital’s political strategy with its other top managers (Director General, facility E).  Discuss the care of certain patients with other professionals in the department: doctors, Nurse managers or secretaries (Head of Unit, facility E).  Contact a social worker about a patient (Head of Unit, facility E).  Contact the general practitioner of patients who are being discharged (Head of Unit, facility E).  Answer the phone and manage the admission of patients requiring hospitalisation (Head of Unit, facility E).  Share ideas with other executives during a meeting (Director, facility G).  Meet with the Assistant to the Director of Human Resources to discuss various ongoing issues (Director, facility G).  Collaborate with the person responsible for finding replacements for absent nurses (Nurse manager, facility G). | | | | |
| Set an example |  | Participate in consultative bodies and working groups  Provide a substitute or alternative, be available during rest periods or unplanned absences of a caregiver  Apply safety rules and good practices  Respond to patients' and families' solicitations | | | |  | | Participate in meetings of the facility's quality and safety bodies such as the Medical Committee (Director General and Head of Unit, facility E).  Attend meetings of the facility’s quality and safety committees, such as the Food and Nutrition Liaison Committee, and the weekly medico-administrative staff meeting (Director and Nurse manager, G).  Participate in the facility’s quality and safety management committees (Director of Nursing and Nurse manager, facility F).  Unexpectedly, temporarily replace a nurse manager from another department, and then a nurse from her department (Nurse manager, facility F).  Wash hands with a hydroalcoholic solution (Nurse manager, facility B).  Wash hands regularly with a hydroalcoholic solution before entering and leaving a patient’s room (Nurse manager and Director of Nursing, facility C).  Ensure the traceability and archiving of safety data that had not been archived (equipment maintenance order) (Nurse manager, facility C).  Put away a cot that was obstructing a corridor (Nurse manager, facility D).  Stop in the hallway to offer assistance to the relative of a patient who is clearly lost (Director of Nursing, facility D).  Support the family member of a patient who had just collapsed in the emergency department (Director of Nursing, facility B). | | | | |
| Direct safety policy and its implementation (make decision) |  | | Define values and objectives for the facility  Make trade-offs and validate, usually by working with others, the multitude of decisions taken independently by various managers (like the conductor of an orchestra)  Allocate resources (define staffing levels, the purchase of care systems, manage budgets) | | |  | Define the procedure to be followed for patient admissions and discharges (Director of Nursing, facility B).  Validate proposals for patient information documents (Director of Nursing, facility B).  Organise rotas for the department’s personnel, the distribution of staff in rooms and the scheduling of interventions according to the patient’s history and needs (Nurse manager, facility C).  Confirm the recruitment of a candidate for a nursing post, to compensate for the absence of a staff nurse. The candidate was selected by the Nurse manager of the department concerned (Director of Nursing, facility D).  Define corrective actions to be implemented within the establishment following an adverse event (ask the establishment’s IT department to train doctors in the use of a software program and develop a user procedure agreed by all doctors) (Director, facility G).  Draw up a new patient admission procedure with the Director of Nursing (Nurse manager, facility G)  Establish job descriptions for professionals under his/ her authority from the perspective of a self-assessment audit by medical and paramedical professionals (Director of Nursing, facility F). | | | | |  |
| Plan |  | |  | | |  | Manage the planning of temporary operating theatre staff (Director of Nursing, facility D).  Manage rotas for the department’s paramedical staff and midwives, taking into account holidays, time off for managers, sick leave, unforeseen absences, etc. (Nurse manager, facility D).  Manage beds and patients, i.e., physically block access to certain rooms in a department about to be renovated and move patients scheduled to occupy these rooms to other rooms (Nurse manager, facility D).  Manage the replacement of a nurse on sick leave (Nurse manager, facility G).  Organise the rota for paramedical staff so that everyone can attend an institutional meeting (Nurse manager, facility F). | | | | |  |
| Interacting with outsiders |  | |  | | |  | Contact a temp agency to recruit staff to compensate for the absence of a nurse at the weekend (Nurse manager, facility B).  Contact a volunteer association in the context of palliative care (Nurse manager, facility B).  Welcome and help a maintenance subcontractor working in the endoscopy department (Nurse manager, facility C).  Recruit. Welcome a candidate for a nursing position (Nurse manager, facility F).  Respond to a request from police and discuss with a representative of the Medical Association the disappearance of a patient several months earlier (Director, facility G). | | | | |  |
| Manage conflict |  | |  | | |  | Meet a patient who was dissatisfied because he wanted a single room, and another who had been refused a stay in follow-up care by a doctor (Nurse manager, A).  Manage the case of a healthcare professional who had a problem with his unit’s manager (Director of Nursing, A). | | | | |  |
| Maintain professional clinical practice |  | |  | | |  | Carry out consultations (Doctor, Head of Division, facility E.) | | | | |  |
|  | | | | | | | | | | |  |  |
